# Characterization of metapopulation of *Ellobium chinense* through Pleistocene expansions and four covariate *COI* guanine-hotspots linked to G-quadruplex conformation

**DOI:** 10.1038/s41598-021-91675-5

**Published:** 2021-06-10

**Authors:** Cho Rong Shin, Eun Hwa Choi, Gyeongmin Kim, Su Youn Baek, Bia Park, Jihye Hwang, Jumin Jun, Hyun Jong Kil, Hyunkyung Oh, Kyungjin Lee, Sa Heung Kim, Jongrak Lee, Seung Jik Suh, Dong-min Park, Ho Young Suk, Yong Seok Lee, Young Sup Lee, Ui Wook Hwang

**Affiliations:** 1 Department of Biology Education, Teachers College and Institute for Phylogenomics and Evolution, Kyungpook National University, Daegu, 41566 South Korea; 2 School of Life Science, Graduate School, Kyungpook National University, Daegu, 41566 South Korea; 3 Biological and Genetic Resources Utilization Division, National Institute of Biological Resources, Incheon, 22689 South Korea; 4 Institute of the Sea Life Diversity, In the Sea, Incheon, 63573 South Korea; 5 Byunsanbando National Park Office, Korea National Park Service, Buan-gun, Jeonrabuk-do 56342 South Korea; 6 Department of Life Sciences, Yeungnam University, Gyungsan, Gyeongsangbuk-do 38541 South Korea; 7 Department of Life Science and Biotechnology, College of Natural Sciences, Soonchunhyang University, Asan, Chungcheongnam-do 31538 South Korea; 8 Institute for Korean Herb-Bio Convergence Promotion, Kyungpook National University, Daegu, 41566 South Korea; 9 School of Industrial Technology Advances, Kyungpook National University, Daegu, 41566 South Korea

**Keywords:** Ecology, Evolution

## Abstract

The land snail *Ellobium chinense* (L. Pfeiffer, 1855) (Eupulmonata, Ellobiida, Ellobiidae), which inhabits the salt marshes along the coastal areas of northwestern Pacific, is an endangered species on the IUCN Red List. Over recent decades, the population size of *E. chinense* has consistently decreased due to environmental interference caused by natural disasters and human activities. Here, we provide the first assessment of the genetic diversity and population genetic structures of northwestern Pacific *E. chinense*. The results analyzed with *COI* and microsatellites revealed that *E. chinense* population exhibit metapopulation characteristics, retaining under the influence of the Kuroshio warm currents through expansion of the Late-Middle and Late Pleistocene. We also found four phylogenetic groups, regardless of geographical distributions, which were easily distinguishable by four unidirectional and stepwise adenine-to-guanine transitions in *COI* (sites 207–282–354–420: A–A–A–A, A–A–G–A, G–A–G–A, and G–G–G–G). Additionally, the four *COI* hotspots were robustly connected with a high degree of covariance between them. We discuss the role of these covariate guanines which link to form four consecutive G-quadruplexes, and their possible beneficial effects under positive selection pressure.

## Introduction

**Figure 1:**
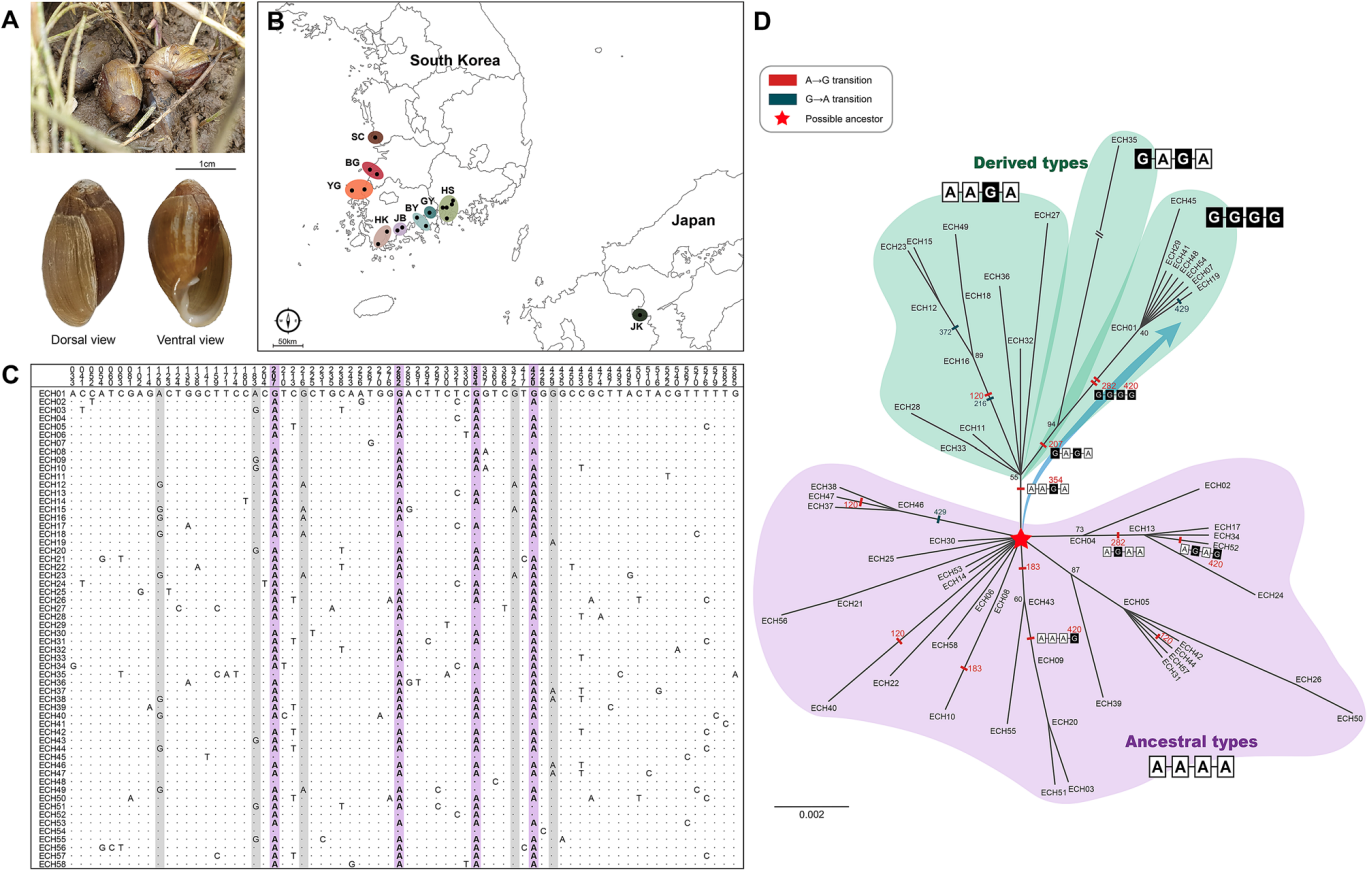
Photographs, sampling localities, polymorphic sites inferred from *COI* haplotypes, and an unrooted maximum likelihood tree for *Ellobium chinense* inhabiting the northwestern Pacific coast (South Korea and Japan). (**A**) A salt grass habitat of *E. chinense* (Hadong, Gyeongnam, South Korea; taken in July 2020), and dorsal and views of the snail’s shell. Refer to Fig. S1 for the full landscape of the habitats. The scale bar indicates 1 cm. (**B**) The collection sites of *E. chinense* (marked with black dots) grouped into nine possible populations (colored circles). Full details are given in Table 1. (**C**) Alignment of 71 polymorphic sites inferred from the alignment of 58 *COI* haplotype sequences (Fig. S2) from 140 *E. chinense* individuals; 31 sites are parsimoniously informative. The four light pink columns indicate the four unidirectional stepwise A → G transition hotspots, which could be key sequences for haplotype classification. The five sites partially colored with light aquamarine are additional A → G transitions, with a few exceptional G → A transitions. Overall, guanine-rich *COI* sequences were evident, likely were caused by unidirectional, biased, A → G transitions during the evolution of *E. chinense*. (**D**) An unrooted maximum likelihood tree reconstructed with 58 *COI* haplotypes. Four different genetic groups are represented by four guanine hotspots: A–A–A–A, A–A–G–A, G–A–G–A, and G–G–G–G. These are depicted with white boxes for A and black boxes for G. The tendency for unidirectional, stepwise, A → G transitions from a plausible ancestor, A–A–A–A (pink star), to the most derived type, G–G–G–G, are shown with a large blue arrow. The additional A → G transitions and exceptional G → A transitions are marked with red and blue boxes, respectively. Node confidence values near nodes are depicted with bootstrapping values in percent. Refer to Fig. S3 for a rooted maximum likelihood tree. The photos and pictures were edited using Adobe Photoshop v.22.2 and Adobe Illustrator v.25.2 (https://www.adobe.com). The basic map is from a free map providing site (https://d-maps.com), which is modified with Adobe Illustrator v.25.2.

*Ellobium chinense* (L. Pfeiffer, 1855)^1^, of the family Ellobiidae (Gastropoda, Eupulmonata, Ellobiida), is a small, air-breathing, land snail that dwells between-stones in freshwater tidelands near the shoreline and on halophytes such as *Zoysia sinica*, as shown in Figs. 1A and S1. Its distribution range is confined only to the northwestern Pacific coasts including the coastlines of South Korea, Japan, and China^2^. Importantly, *E. chinense* is a key indicator of the environmental health of the intertidal zone of its habitat. However, over recent decades, its natural habitat and the number of populations and individuals have rapidly declined due to environmental stresses, such as typhoons and tsunamis, and human activities including land reclamation and coastal development; thus, *E. chinense* has been designated as an endangered species on the South Korean Regional Red List^3^, as a vulnerable species on the Japanese Regional Red List, and as an endangered species on the International Union for Conservation of Nature (IUCN; https://www.iucn.org).

Sufficient genetic diversity is important for ensuring the persistence of species, increasing their adaptive potential in the face of environmental change, and avoiding inbreeding depression^4^. On the other hand, the loss of intraspecific genetic diversity dramatically reduces the capacity for species to respond to novel selection pressure and thereby increases their extinction risk^5^. In recent decades, research on the population genetics of endangered metazoan species has been increasingly conducted because of its central importance in planning both in-situ and ex-situ conservation efforts^6^. In such research, molecular markers, including specific mitochondrial genes [mainly cytochrome *c* oxidase I (*COI*)]^7^, whole mitochondrial genomes^8^, and microsatellites^9^ are consistently used. Given the importance of determining the genetic diversity and population genetic structure of an endangered species in conservation planning, it is surprising that such research has rarely been done with northwestern Pacific *E. chinense*.

In the present study, therefore, we provide the first assessment of genetic diversity among populations of northwestern Pacific *E. chinense* (sampled in South Korea and Japan). Our analyses were based on the popular DNA barcode region of the mitochondrial gene *COI* and 10 selected microsatellite markers. In total, 140 *COI* data (consisting of 139 South Korean samples and one Japanese sample), and 54 microsatellite data (from South Korea only) were analyzed. In addition, we employed four distinct covariate adenine/guanine hotspots observed on *COI* which are distinct key sequences to easily classify phylogenetic groups of *E. chinense*; these sites were closely associated with G-quadruplex structure conformation. G-quadruplexes, i.e., four-stranded noncanonical DNA structures, can be spontaneously formed by Hoogsteen bonds between stacked sets of four guanines from each of four separated runs of two, three, or four guanines^10^. A stable G-quadruplex within the coding sequences (CDS) causes ribosome stalling; protein expression can be enhanced through silent mutations that affect its stability^11^. Herein, by determining the genetic diversity and structures of populations of *E. chinense*, we were able to discuss not only the roles of the four covariate hotspots on the CDS of *COI* in relation to G-quadruplex structure conformation but also the associated beneficial and resistant effects against environmental perturbation such as oxidative stress.

## Results

### Genetic diversity of *E. chinense* based on COI

The partial fragment of *COI*, 595 bp in length, was sequenced from 113 *E. chinense* individuals collected from the eleven sites in South Korea (Table 1). The resultant *COI* sequences were aligned together with 27 *COI* individual sequences^12–14^ retrieved from the NCBI GenBank (Table 1). The latter consists of 26 from six collection sites in South Korea and one from a Japanese site. Hence, 140 *COI* sequences of *E. chinense* were analyzed, representing 18 collection sites at the nine populations in South Korea and Japan (Table 1). Based on the alignment set (no indels) of these 140 *COI* sequences (Data S1), we obtained a total of 58 *COI* haplotypes, of which 43 were singleton, appeared in only a single site. The novel 41 out of 58 *COI* haplotypes obtained were registered under the GenBank accession nos. MW265437–MW265477 (Table S2). According to the sequence alignment of the 58 *COI* haplotypes (Fig. S2; Data S2), there were 71 polymorphic sites and 31 parsimoniously informative sites (Fig. 1C), among which four adenine/guanine hotspots at 207, 282, 354, and 420 were ascertained to articulately divide the haplotypes of *E. chinense* into four meaningful phylogenetic groups: (a) A(207)–A(282)–A(354)–A(420), (b) A–A–G–A, (c) G–A–G–A, and (d) G–G–G–G.

**Table 1:** List of collection sites and the number of individuals of *Ellobium chinense* with genetic markers applied to each of the nine populations in South Korea and Japan.

Populations	Collection sites	N	Genetic markers		References
			*COI*	Microsatellites	
SC	Seo-myeon, Seocheon-gun, Chungnam, South Korea	5	**√**		Yi et al*.*^12^
BG	Byeonsan-myeon, Buan-gun, Jeonbuk, South Korea	**16**	**√**	**√**	**This study**
	Simwon-myeon, Gochang-gun, Jeonbuk, South Korea	5	**√**		Yi et al*.*^12^
YG	Nagwol-myeon, Yeonggwang-gun, Jeonnam, South Korea	**10**	**√**		**This study**
	Hongnong-eup, Yeonggwang-gun, Jeonnam, South Korea	**19**	**√**		**This study**
HK	Chillyang-myeon, Gangjin-gun, Jeonnam, South Korea	**11**	**√**	**√** (10)^a^	**This study**
	Bukpyeong-myeon, Haenam-gun, Jeonnam, South Korea	5	**√**		Yi et al*.*^12^
JB	Anyang-myeon, Jangheung-gun, Jeonnam, South Korea	1	**√**		Kang et al*.*^13^
	Hoecheon-myeon, Boseong-gun, Jeonnam, South Korea	**1**	**√**		**This study**
BY	Beolgyo-eup, Boseong-gun, Jeonnam, South Korea	**10**	**√**		**This study**
	Hwayang-myeon, Yeosu-si, Jeonnam, South Korea	5	**√**		Yi et al*.*^12^
GY	Gwangyang-eup, Gwangyang-si, Jeonnam, South Korea	**9**	**√**		**This study**
HS	Seopo-myeon, Sacheon-si, Gyeongnam, South Korea	**10**	**√**		**This study**
	Chukdong-myeon, Sacheon-si, Gyeongnam, South Korea	**9**	**√**		**This study**
	Yonghyeon-myeon, Sacheon-si, Gyeongnam, South Korea	5	**√**		Yi et al*.*^12^
	Jingyo-myeon, Hadong-gun, Gyeongnam, South Korea	**10**	**√**	**√**	**This study**
	Idong-myeon, Namhae-gun, Gyeongnam, South Korea	**8**	**√**	**√**	**This study**
JK	Oita Prefecture, Kyushu Island, Japan	1	**√**		Romero et al.^14^
**Total**		**140**			

‘N’ indicates the number of individuals.

^a^Only ten samples out of 11 were employed.

The bold letters indicate the data obtained from the present study.

Based on the *COI* haplotype sequence alignment (Fig. S2; Data S2), we reconstructed a ML tree using *Ellobium aurisjudae* as an outgroup. In the resultant tree topology (Fig. S3), it was confirmed that *E. chinense* appeared as a monophyletic group, but no distinction between the haplotypes from each geographical population was observed. To define detailed relationships among the *COI* haplotypes, the outgroup was removed and then an unrooted ML tree (Fig. 1D) was reconstructed. The resultant tree showed two distinctive phylogenetic groups, namely A–A–A–A and the other groups (including at least one G or more in the four positions), regardless of collection localities. The A–A–A–A group included 35 of the 58 *COI* haplotypes. The others could be divided into the A–A–G–A group (*N* = 12: ECH11, 12, 15, 16, 18, 23, 27, 28, 32, 33, 36, and 49), the G–A–G–A group (*N* = 1: ECH35), and the G–G–G–G group (*N* = 8: ECH01, 07, 19, 29, 41, 45, 48, and 54).

As shown in Table S2 and Fig. S3, ECH01 was a dominant member of the G–G–G–G group with the most individuals (27), which appeared across all the South Korean populations examined here. As shown in Fig. 1D, the A–A–A–A group is likely to be an ancestral type because it was most frequently found in the other species within Ellobiidae (unpublished data) and its haplotype diversity was the highest among the four genetic groups. Given that the G–G–G–G group exhibited much lower haplotype diversity than the A–A–A–A group, and was not observed in any other ellobiid species (unpublished data), it is reasonable to suggest that the G–G–G–G group is a derived rather than an ancestral type. Thus, as shown in Fig. 1D, it is conceivable that unidirectional and stepwise A → G transition events from A–A–A–A to G–G–G–G may have been occurred in *E. chinense*. Within the A–A–A–A and A–A–G–A groups, parsimoniously informative A → G transition events were found at the sites 120 (ECH12, 15, 16, 18, 23, 38, 40, 44, and 49) and 183 (ECH3, 9, 10, 20, 43, 50, and 55), with a few exceptional cases of G → A at the sites 216 (ECH12, 15, 16, 18, and 23), 372 (ECH12, 15, and 23), and 429 (ECH37, 38, 46, and 47; ECH19 found in the G–G–G–G group).

As indicated in Table 2, the nucleotide diversity (π) is relatively low among the nine populations of *E. chinense*, ranging from 0.00749 (population BG) to 0.01042 (SC) with an average of 0.00865, whereas the haplotype diversity was very high across these populations, ranging from 0.924 (YG) to 1.000 (SC and JB) with an average of 0.939). All values of Tajima's *D* and Fu's *F*_S_ were congruently negative, with averages of − 1.87100 (P < 0.05) and − 2.75886 (not statistically significant), respectively, indicating that the signature of demographic expansions recently occurred in *E. chinense*.

**Table 2:** The result summary of genetic diversity analyses and neutrality tests performed with 140 *COI* sequences from *Ellobium chinense* along the nine populations in South Korea and Japan.

Populations	N	N_h_	N_p_	*h*	π	S	k	Tajima’s *D*	Fu’s *F*s
SC	5	5	3	1.000	0.01042	13	6.200	− 0.04655	− 1.01140
BG	21	15	4	0.938	0.00749	22	4.457	− 1.02706	− 6.82701**
YG	29	16	10	0.924	0.00912	31	5.429	− 1.13813	− 4.06280*
HK	17	14	7	0.971	0.00882	25	5.250	− 1.16767	− 6.54343**
JB	2	2	0	1.000	0.00840	5	5.000	nd	nd
BY	15	9	3	0.933	0.00791	17	4.705	− 0.29535	− 1.29300
GY	9	7	4	0.944	0.00952	17	5.667	− 0.45784	− 0.94084
HS	42	21	11	0.926	0.00890	37	5.296	− 1.33327	− 6.84025**
JK (Japan)	1	1	nd	nd	nd	nd	nd	nd	nd
Total/mean	140	58	42	0.939	0.00865	70	5.137	− 1.88691*	− 2.75886

Diversity parameters are given for each locality: N = the number of *CO1* sequences (individuals), N_h_ = the number of haplotypes, N_p_ = the number of private haplotypes, *h* = haplotype diversity, π = Jukes-Cantor corrected estimates of nucleotide diversity, S = the number of segregation sites, K = the average number of pairwise nucleotide differences, and ‘nd’ = not determined. Statistically significant values are written in bold: *P < 0.05 and **P < 0.01. The localities of the populations refer to Table 1 and Fig. 1.

### Population genetic structure of *E. chinense* based on COI

As shown in Table S3, the pairwise *F*_ST_ values between nine *E. chinense* populations were highly low and statistically non-significant, ranging from − 0.21177 (JB and GY) to 0.09091 (JB and JK), indicating the lack of genetic differences among populations. Analysis of molecular variance (AMOVA) for the nine populations of *E. chinense* in South Korea and Japan (Table S4) revealed that 101.05% of the variance was allocated into the level of individuals within each population, while differentiation between populations (− 1.05%) did not appear to contribute to the overall variance.

Upon the results of TCS network analysis, all the *COI* haplotypes were connected by forming a single, large network, conforming that no distinct genetic differences existed among populations (Fig. S4a). The G–G–G–G group, with its representative member ECH01, was located rather at a distal position on the TCS network, which implies that these haplotypes may have recently emerged during the expansion of South Korean *E. chinense* populations. The phylogenetic network analysis (Fig. S4b) also indicated that distinct genetic differences representing the geographical distributions of *E. chinense* did not exist. Indeed, all haplotypes were grouped into a single broad cluster with overlaps between populations, which is in agreement with the results of the TCS network analysis (Fig. S4a). In addition, the mismatch distribution analysis showed a clearly unimodal curve (Fig. S4c), evidently implying the metapopulation of *E. chinense* that may have experienced a recent demographic expansion^15,16^ or through a range expansion with high levels of migration between neighboring demes^17,18^.

**Figure 2:**
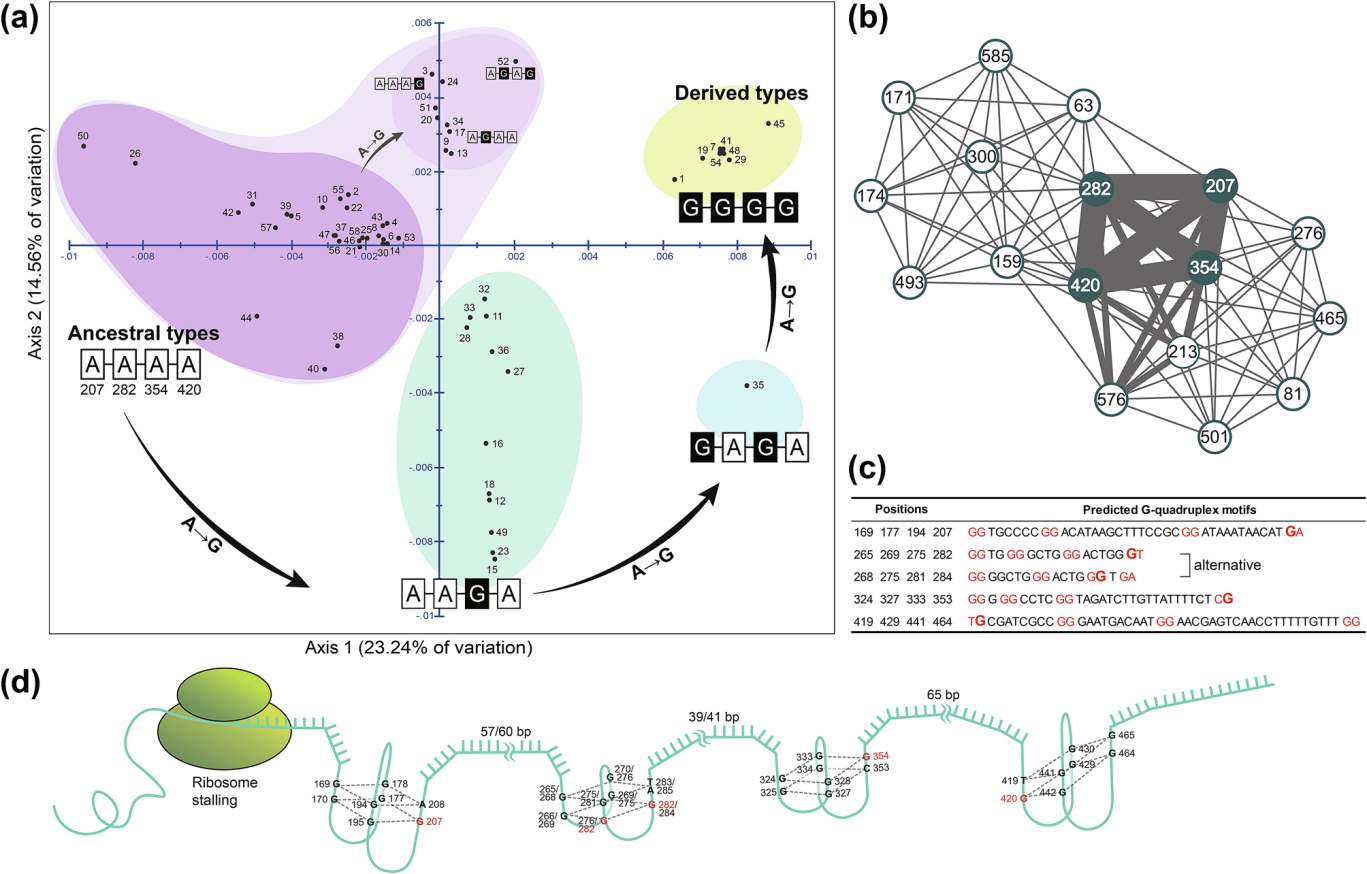
Results of principal coordinate analysis (PCoA), covariance of the four guanine hotspots, and plausible G-quadruplex motifs inferred from 5*8 COI* haplotypes found in northwestern Pacific (mainly South Korean) *Ellobium chinense*; hypothetical ribosome stalling caused by four plausible consecutive G-quadruplex structures in the mRNA of *E. chinense COI*. (**a**) Results of PCoA. Representation of the scores on the first two axes (Axis 1 = 23.33%; Axis 2 = 14.68%) from the matrix of genetic distances estimated with the 58 *COI* haplotypes. Refer to Table 1 for the number of individuals along with the collection site references of the haplotypes; refer to Figs. 1C and S2 for sequence alignment of the 58 *COI* haplotypes. The colored circles indicate four or five genetic groups regardless of geographical distributions. The black arrows indicate unidirectional, stepwise, A → G transitions (**b**) Covariance test results for the four adenine/guanine hotspots that may be linked to the four guanine hotspots inferred from 58 *COI* haplotypes shown in Figs. 1C and S2. In addition, a node in the network represented the position of each sequence, a link represented a covariance position, and the link thickness represented covariance frequency. The detailed data are listed in Data S3. (**c**) Plausible selected G-quadruplex motifs linked to the four adenine/guanine hotspots on *COI*. For the 282-site-related motif, the two alternatives are suggested. Refer to Data S3 for the raw data from the G-quadruplex sequence motif search in *COI*. (**d**) Hypothetical ribosome stalling in the *COI* translation process caused by four consecutive G-quadruplex structures with intervals from 39 to 65 bp. Each structure consists of two stacked G-quadruplexes. For the 282-site-linked G-quadruplex structure, two alternative structures are depicted based on the two alternative motif sequences shown in (**c**). The pictures were edited using Adobe Illustrator v.25.2 (https://www.adobe.com).

The PCoA shown in Fig. 2a provided additional evidence that, regardless of the geographical distribution of South Korean*E. chinense*, there was consistent support for the existence of four distinct phylogenetic groups, i.e., A–A–A–A, A–A–G–A, G–A–G–A, and G–G–G–G (as also shown in Fig. 1). In this analysis, the plausible ancestral A–A–A–A group (with the highest degree of haplotype diversity) was placed mainly around the bottom of the second quadrant, whereas the A–A–G–A group was in the left of the fourth quadrant, the G–A–G–A group (ECH35 only) in the right middle of the fourth quadrant, and the G–G–G–G group (represented by ECH01) was in the first quadrant. The three variation types within the A–A–A–A group, namely A–A–A–G, A–G–A–A, and A–G–A–G, appeared in the ML tree (Fig. 1D; Fig. S3) and were spatially separated from the remaining orthodox members of A–A–A–A, which were located along the Y axis between the first and second quadrants. Based on the results of PCoA (Fig. 2a) and the ML tree (Fig. 1A), we confidently suggest that the four phylogenetic groups of *E. chinense* arose as a result of serial, stepwise A → G transition events in the following order: A–A–A–A (ancestral) → A–A–Ğ–A → Ğ–A–Ğ–A → G–Ğ–G–Ğ (derived). The other local A → G transition events occurred within the A–A–A–A group (A–A–A–Ğ, A–Ğ–A–A, and A–Ğ–A–Ğ) (Fig. 1D; Fig. S3); these could be considered the fifth spatially separated phylogenetic group of *E. chinense*, based only on PCoA (Fig. 2a), being located between the phylogenetic groups G–G–G–G and A–A–A–A but apart from A–A–A–A.

### Covariance of four COI hotspots linked to the conformation of four G-quadruplexes

Similar sequence interval lengths existed between the four adenine/guanine hotspots of *COI* characterized by unidirectional A → G transitional changes: G-(75 bp)–G-(72 bp)–G-(66 bp)–G. Additionally, the covariance analysis indicated that there was a high degree of covariance among these (see the four thick and bold linkage lines in Fig. 2b): out of 58 haplotypes, 44 were covariate between 207 and 282 sites, 44 between 207 and 420 sites, 41 between 282 and 420 sites, 37 between 207 and 354 sites, 32 between 282 and 354 sites, and 32 between 282 and 420 sites (Data S3 in detail). Given the similar intervals and covariance characteristics, we used QGRS-Conserve to search for sequence motifs that could possibly indicate the formation of G-quadruplex structures in relation to the four hotspots on the CDS of the *COI* barcoding region. Our results revealed possible sequences associated with the conformation of four different G-quadruplexes represented by each of the four hotspots (Fig. 2c). The first hotspot located at site 207 may be involved in forming a G-quadruplex structure such as G_2_–N_6_–G_2_–N_15_–G_2_–N_11_–**Ğ**A; the second hotspot at site 282 may be involved in forming G_2_–N_2_–G_2_–N_5_–G_2_–N_5_–**Ğ**T or G_2_–N_2_–G_2_–N_4_–**Ğ**_2_–N_1_–GA; the third hotspot at site 354 may be involved in forming G_2_–N_1_–G_2_–N_4_–G_2_–N_18_–C**Ğ**; and the fourth hotspot, found at site 420, may be involved in the formation of T**Ğ**–N_8_–G_2_–N_10_–G_2_–N_21_–CG.

G-Quadruplex structures generally have 1–7 nucleotides between the four guanine-tracks each consisting of two or three guanines, e.g., G_2–3_–N_1–7_–G_2–3_–N_1–7_–G_2–3_–N_1–7_–G_2–3_; two (for G_2_) or three (for G_3_) G-quadruplexes can form a stacked structure. Considering the typical features of G-quadruplex structures, each of the putative motifs searched for G-quadruplex structure formation is likely to exhibit two G-quadruplexes stacked together (Fig. 2c,d). However, the number of nucleotides between the G-tracks is much larger than usual for some sites, and some G tracks consist of only one G (not two or three). Thus, the G-quadruplex structures formed from the putative motifs are expected to be relatively weak. Assuming that all four putative motifs found in our analyses are capable of forming G-quadruplex structures, the four consecutive G-quadruplexes with intervals of 57/60, 39/41, and 65 bp in order might play a role in reducing or inhibiting transcription or translation of *COI* expression, i.e., they may be an exemplar of downregulation of gene expression; the ribosome stalling process (Fig. 2d).

### Genetic diversity of *E. chinense* inferred from microsatellite markers

**Figure 3:**
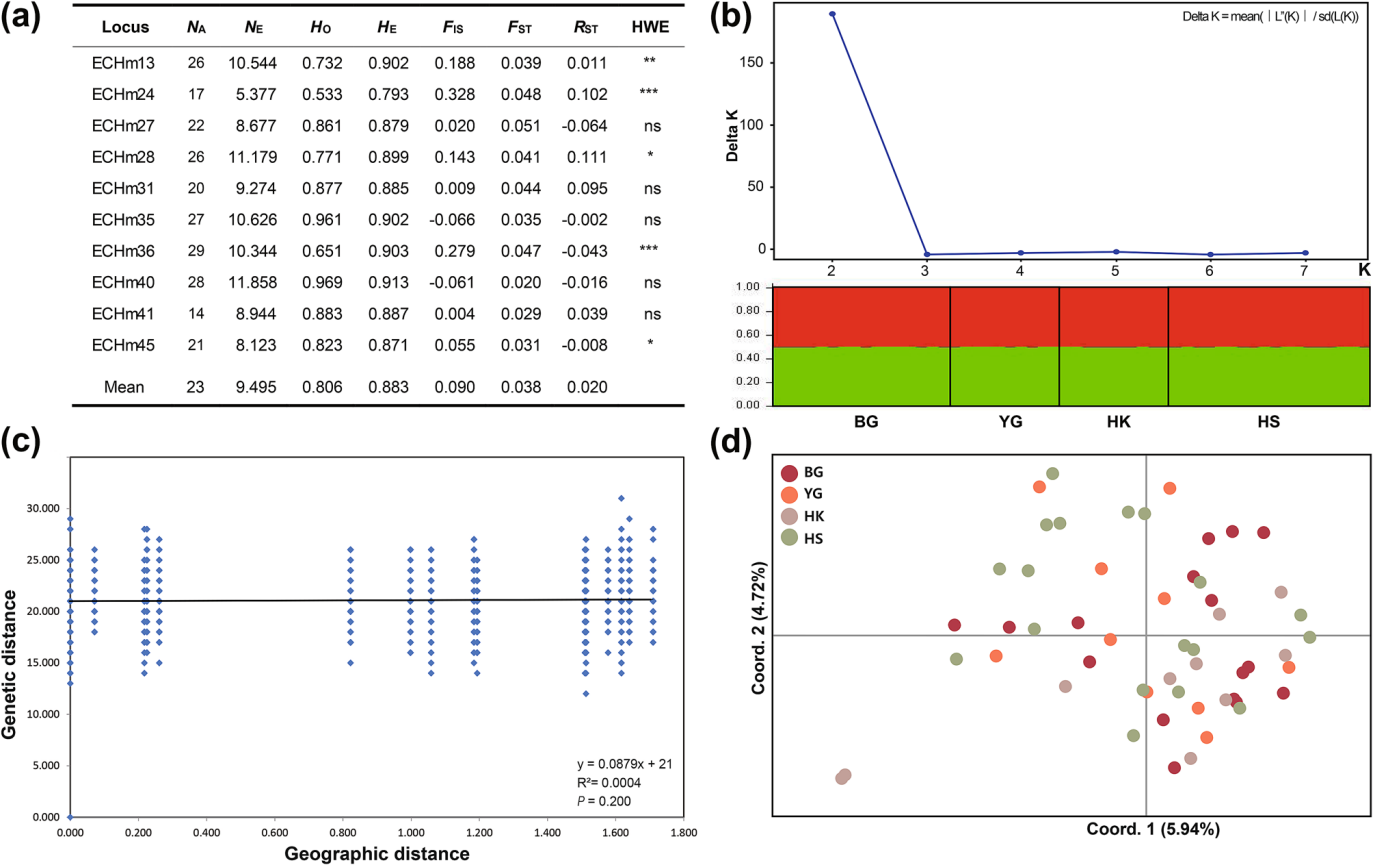
Genetic diversity, population genetic structure, isolation by distance (IBD), and principal component analysis (PCoA) based on 10 selected microsatellite markers and 54 *Ellobium chinense* individuals collected from South Korea. (**a**) Summary statistics for the genetic diversity of *E. chinense*. *N*_*A*_: mean number of alleles, *N*_*E*_: number of effective alleles, *H*_*O*_: observed heterozygosity, *H*_*E*_: expected heterozygosity, *F*_*IS*_: inbreeding coefficient (indicates populations with heterozygote deficit); HWE: loci showing a significant departure from Hardy–Weinberg equilibrium with a global test at the 5% level after a sequential Bonferroni correction (*P < 0.05, **P < 0.01, ***P < 0.001, ns: not significant). (**b**) Determination of the true number of genetic clusters (*K* = 2) by Bayesian clustering using the method of Evanno et al.^16^. *K* statistics are based on the rate of change in the log probability of data between successive *K* values. Bayesian clustering results were obtained using the STRUCTURE program^48^. Refer to Table 1 for information on the four populations: BG, YG, HK, and HS. (**c**) IBD test results showing that genetic distances are not correlated with geographical distances among the sample collection sites. (**d**) A two-dimensional plot from the PCoA results showing that any distinct genetic differences did not exist among the four populations of *E. chinense* in South Korea. The percentage variation attributable to the three principal coordinate axes was 10.66% (Axis 1 = 5.94%; Axis 2 = 4.72%).

The ten polymorphic microsatellite loci were amplified from 54 individuals collected from the four populations (BG, YG, HK, and HS) of *E. chinense* in South Korea (Table 1), and the PCR products were then sequenced and analyzed to elucidate the level of genetic diversity of *E. chinense*. Figure 3a shows the microsatellite diversity indices related to genetic diversity for the four *E. chinense* populations. The number of observed alleles per locus (*N*_A_) varied greatly among loci, from 14 (ECHm41) to 29 (ECHm36). The number of effective alleles per locus (*N*_E_) ranged from 5.377 (ECHm24) to 11.858 (ECHm40), with the mean of 9.495. Overall, the effective number of alleles (*N*_E_ = 9.495) was smaller than the mean number of alleles (*N*_A_ = 13.4). The expected mean heterozygosity (*H*_E_) per locus ranged from 0.793 (ECHm24) to 0.913 (ECHm40), while the average value was 0.883. The observed heterozygosity per locus (*H*_O_) was from 0.533 (ECHm24) to 0.969 (ECHm40), with a mean value of 0.806. Thus, the expected heterozygosity (*H*_E_ = 0.883) was larger than the observed heterozygosity (*H*_O_ = 0.806). The average of the genetic differentiation coefficient *F*_ST_ was estimated relatively low to be 0.038; it ranged from 0.020 (ECHm40) to 0.051 (ECHm27). Table S5 provides more detail on the genetic diversity estimates within each of the four populations (BG, YG, HK, and HS) of *E. chinense*.

### Population genetic structure of *E. chinense* based on microsatellite markers

According to Table S6, the estimated *F*_ST_ values, showing the degree of genetic differentiation among pairs of populations, were statistically significant (P < 0.05) only between the populations BG and HS (*F*_ST_ = 0.120) and HK and HS (*F*_ST_ = 0.0184). In contrast, pairwise *R*_ST_ was not significant among populations. Generally, pairwise genetic differentiation among populations was low, indicating that the *E. chinense* populations were not genetically differentiated from each other.

The distributions of molecular genetic variation among and within populations, as estimated by AMOVA, are shown in Table S7; only 0.85% of the total molecular variation was attributed to interpopulation differentiation, whereas 86.40% of the variation was within populations. This supports the conclusion that significant genetic differences did not exist among the four examined *E. chinense* populations.

Based on microsatellite markers, the 54 *E. chinense* individuals were further examined to determine population stratification. The optimal number of clusters, *K,* according to the method of Evanno et al.^19^, was 2 (Fig. 3b). However, with this method, it is only possible to infer with confidence about clusters ≥ 2. Indeed, the LnP (D) plot shows a strong drop off in model fit only after *K* = 2 (data not shown); thus, the ancestry proportions were plotted for each individual given two clusters. All individuals were equally admixed (Fig. 3b) across the range, supporting a single genetic cluster. IBD analysis indicated that the pairwise observed genetic differences were not correlated with the pairwise geographical distances among the four examined *E. chinense* populations (Fig. 3c). Consistently, the PCoA results (Fig. 3d) showed that all populations were clustered into a single genetic group. Taken together, the Bayesian clustering approach, IBD analysis, and PCoA provided consistent results that indicated the lack of spatial seperation among the four *E. chinense* populations from South Korea and Japan. These results are coincident with the metapopulation of *E. chinense* formed through recent expansion, as inferred from *COI* haplotypes (Figs. 1 and 2).

## Discussion

**Figure 4:**
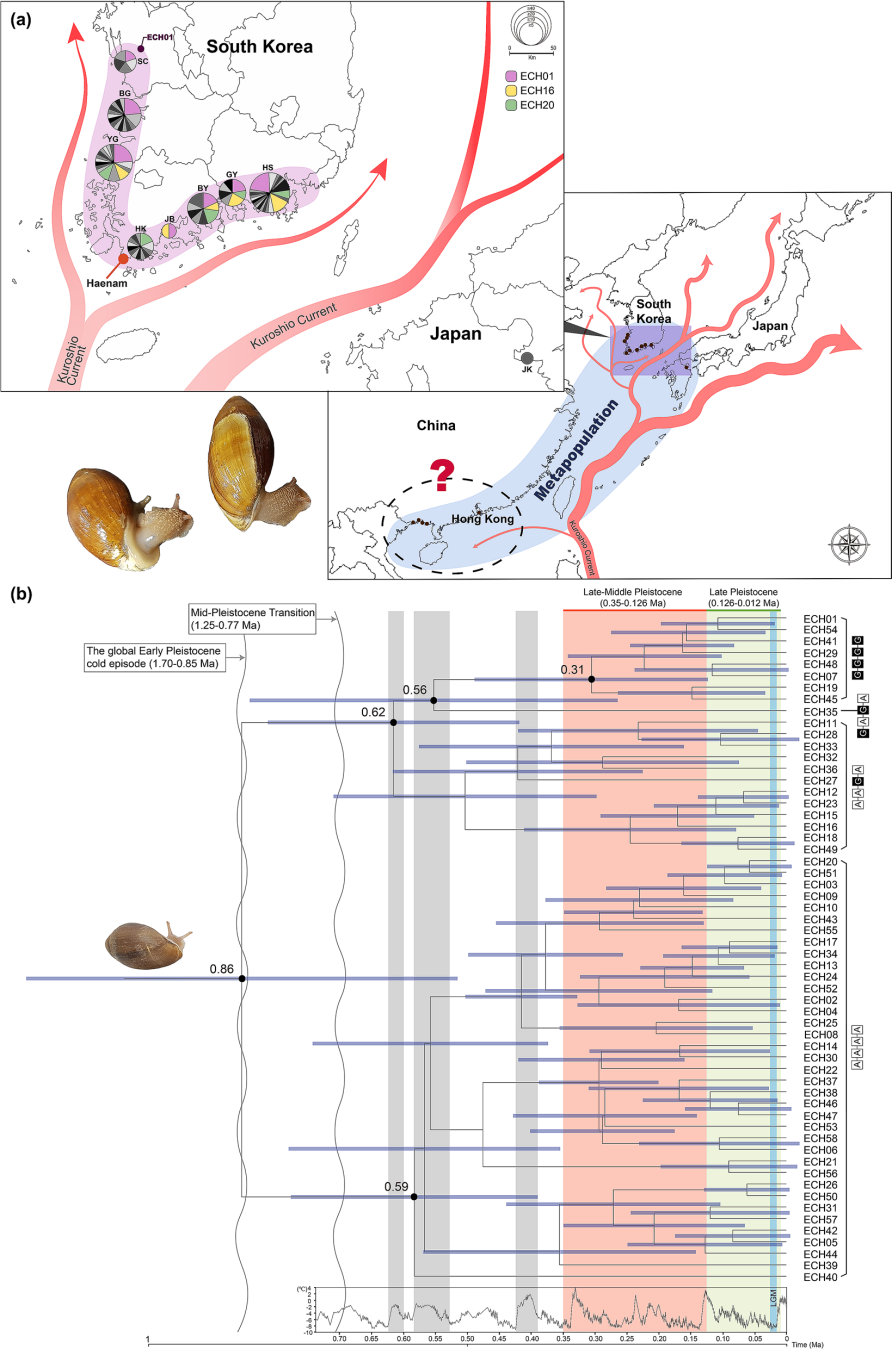
Geographical distribution of 58 *COI* haplotypes from *Ellobium chinense* in South Korea and Japan, and the results of molecular clock analysis using the haplotypes and BEAST 2.6.0. (**a**) Geographical distribution of 58 *COI* haplotypes from *E. chinense* shown on a map of South Korea and Japan. Each haplotype is represented by a pie chart sized in proportion to its frequency in each population. The haplotype ECH01 was observed in all populations. Results show that *E. chinense* from South Korea and Japan exhibit metapopulation dynamics. Given the effects of the Kuroshio warm current, the southern China populations might belong to the metapopulation (but this requires confirmation by further research). (**b**) Molecular clock analysis showing geological times, including the first appearance of the subfamily Ellobiinae (at the Eocene Optimum immediately after the Paleocene–Eocene Thermal Maximum; refer to Fig. S5), the divergence times of the four phylogenetic groups of *E. chinense*, and the periods of explosive population expansion with an increase in *COI* haplotype diversity [Late-Middle and Late Pleistocene prior to the Last Glacial Maximum (LGM)]. The gray columns mark interglacial periods related to haplotype divergences of *E. chinense* before Late-Middle Pleistocene. Light-red and light-green boxes mark the periods of rapid population expansions and the number of bifurcations (= increased haplotype diversity), respectively. The four genetic groups, depicted by A–A–A–A, A–A–G–A, G–A–G–A, and G–G–G–G, are explained in detail in Figs. 1 and 2. The full BEAST analysis results are presented in Fig. S5. Outgroups and the calibration points are described in detail in the section of “Materials and methods”. The photos and pictures were edited using Adobe Photoshop v.22.2 and Adobe Illustrator v.25.2 (https://www.adobe.com).

Over recent decades, population numbers have decreased sharply due to coastal development, tidal flat reclamation, and salt marsh destruction. *E. chinense* is also known to be a group of organisms that are particularly more susceptible to human activities than other counterparts of marine ecosystems^20^. Population genetic analyses of *E. chinense* conducted here based on *COI* and microsatellite markers consistently showed the absence of any distinct genetic differences with which to distinguish populations; this may be attributable to the pelagic larval phase being able to easily cross oceanic dispersal barriers via Kuroshio warm currents^21^. It is most likely that *E. chinense* exhibits typical metapopulation characteristics, which are commonly observed in other marine organisms^22^: (1) high haplotype diversity but low nucleotide diversity, (2) lack of genetic differences among populations, and (3) low genetic differentiation (frequent genetic flows between populations). Given the general metapopulation characteristics, it is reasonable to conclude that *E. chinense* populations inhabiting South Korea and Japan are a single broad metapopulation that has arisen due to repeating extinctions and migrations caused by continuous and rapid changes in environmental and habitat conditions during the Late-Middle and Late Pleistocene (Figs. 4b and S5).

As shown in Fig. 4a, a part of the Kuroshio warm current passing through Japan diverges near Haenam to the south and west coasts of the Korean Peninsula. Thus, it is assumed that *E. chinense* larvae spread east along the southern coast and north along the western coast according to the influence of the Kuroshio warm currents. In summary, our results strongly suggest that South Korean and Japanese populations of *E. chinense* may possess metapopulation characteristics associated with Late-Middle and Late Pleistocene expansions (Fig. 4b), and that all northwestern Pacific *E. chinense* affected by passes of the Kuroshio warm current are likely to be a single, large metapopulation (Fig. 4a). However, our study is limited because we included only one *E. chinense* sample from Japan and did not analyze samples from China, given the difficulties in international collection of endangered species. Therefore, we cannot conclusively state that northwestern Pacific *E. chinense* from the Korean Peninsula, southern Japan, and southern China exhibit overall metapopulation dynamics. Indeed, further research is required in which additional *E. chinense* samples from southern Japan and southern China are collected and comprehensive analyzed.

According to the results of our molecular clock analysis, as shown in Fig. 4, the A–A–Ğ–A group diverged from the ancestral type A–A–A–A 0.85 Ma, Ğ–A–Ğ–A from A–A–Ğ–A 0.61 Ma, and then G–Ğ–G–Ğ from Ğ–A–Ğ–A 0.55 Ma. Considering global temperature related to sea level changes over the past 0.85 mya, it is likely that augmentation of *COI* haplotype diversity, i.e., the number of branch bifurcations, is congruent with interglacial periods exhibiting relatively higher global temperatures and sea levels. The divergence time between the ancestral A–A–A–A group and the other derived groups was estimated at 0.85 Ma, which corresponds to the end of the global early Pleistocene cold episode (1.70–0.85 Ma), which was succeeded by the appearance of warm sea currents in East Asia, the South China Sea, North Pacific Ocean, and Japanese islands^23^. After the Mid-Pleistocene Transition (1.25–0.77 Maa), which is also known as the Mid-Pleistocene Revolution, divergences of Ğ–A–Ğ–A (0.61 Ma) and G–Ğ–G–Ğ (0.55 Ma) also occurred during interglacial periods (EPICA community members, 2004), as shown in Fig. 4b. In particular, explosive augmentation of *COI* haplotype numbers and demographic population expansion, as expected from the negative values of Tajima's *D* and Fu's *F*_S_ neutrality tests in the present study, may have occurred largely during the Late-Middle Pleistocene (i.e., the Late Chibanian Age; 0.350–0.126 Ma) and Late Pleistocene (i.e., the Tarantian Age; 0.126–0.012 Ma), and might have continued to the Last Glacial Maximum (0.027–0.002 Ma) (Fig. 4b).

Intriguingly, we found that four adenine-to-guanine transition hotspots at the 207, 282, 354, and 420 sites of the *COI* haplotype alignment of *E. chinense* could determine the production of genetic groups in the species, regardless of geographical distribution. Based on our PCoA, it seems possible to divide the *COI* haplotypes into five different genetic groups within a single metapopulation of *E. chinense*. The largest group, A–A–A–A, which is plausibly ancestral, includes 35 of the 58 haplotypes; the most derived group, G–G–G–G, consists of only 8 haplotypes. Despite a few G → A transitions, such as at the 216, 372, and 429 sites, purine base transitions seem to be biased to unidirectional A → G transitions, including the four hotspot sites and two other sites, 120 and 183. In particular, the unidirectional A → G transition events are shown in the serial and stepwise transitions of the four hotspots in *COI*. Such a strong unidirectional tendency can produce guanine richness under plausible positive selection pressure. For example, a positive selective bias from adenine-to-guanine transition has recently been characterized in soft-bodied cephalopods by Moldovan et al.^24^. These authors suggested that edited adenines tend to be substituted to guanines, and this tendency is supported by positive selection at highly edited sites. Furthermore, they suggest that such A → G transition bias can yield beneficial effects of increased phenotypic diversity in a low-polymorphic population, which can enhance adaptation, and facilitate the evolutionary process. Similar observations were recently reported in *Drosophila* and human mRNA editing sites^25^. Therefore, guanine-rich DNA sequences, resulting from the serial and stepwise A → G transitions reported here, could increase phenotypic diversity in a low-polymorphic metapopulation such as that of *E. chinense*, which might have enhanced the possibility of adaptation in terrestrial and brackish water areas as the species passed through repeated long glacial and relatively short interglacial periods during the Late-Middle Pleistocene and Late Pleistocene (Fig. 4b).

We found strong covariance among the four guanine hotspot sequences in *COI*, each of which may link respectively to a putative G-quadruplex motif. Guanine-rich sequences are known to be capable of producing noncanonical conformations of G-quadruplexes (also known as G-tetrads or G4) in DNA or RNA sequences^26^. In the nuclear genome, G-quadruplex motifs have been associated with genome instability and gene expression defects; however, they are mainly recognized as regulatory structures that control gene expression or positively affect telomere capping^27^. Recent studies^28^ have reported that G-quadruplex structures can form in the mitochondrial genome and even on the CDS in protein-coding genes^29^. Although G-quadruplexes typically have harmful effects, those formed in mitochondrial genomes can help in the response to certain environmental stresses such as oxidative stress^30^. Deficiency in *COI* within mitochondria caused by G-quadruplexes could result in positive selection bias that reduces the production of reactive oxygen leading to less oxidative damage and a selective advantage during competition with other mitochondria within the same cell, which could generate homoplasmy for *COI* deficiency. Hershman et al.^31^ suggested that cells with cytochrome c oxidase deficiency are apoptosis resistant and therefore more likely to survive. Active *COI* oxidizes cytochrome c, which activates pro-caspase 9 leading to apoptosis. Assuming that the four guanine hotspots link to create four G-quadruplex structures, they may function in the regulation (mainly inhibition) of *COI* expression at the transcription or translation levels, and in modulating (mainly reducing) the concentration of activated *COI* within the mitochondria. This would reduce oxidative damage and increase resistance to apoptosis with lower reactive oxygen production. Such G-quadruplex conformation-related oxidative stress and apoptosis resistance could provide a selective advantage in competition with other mitochondria in a cell. According to a previous study on the energy metabolism of the garden snail *Helix aspersa*, natural selection can reduce energy metabolism in a land snail^32^. Intriguingly, the slow behaviors of land snails that have led to reduced energy consumption might be associated with downregulation of *COI* expression modulated by G-quadruplex motifs on the CDS of *COI* (as we hypothesized; see Fig. 2).

Determining the genetic diversity and population genetic structure of isolated and threatened species is of great importance when planning a suitable conservation strategy^33^, especially for the selection of appropriate populations and management methods aimed at maintaining genetic variation. Highly diverse populations can be targeted for protection while small and depleted populations can be subjected to management actions that restore their diversity. For example, enhancement or reinforcement plans aim to add individuals to existing populations while reintroduction plans aim to re-establishment a species within its natural habitat where it has become extinct^34^. From the present study, the genetic resources of the endangered *E. chinense* could be essential for successfully designing conservation strategies aimed at preserving its population and restoring its habitat. In research into endangered species, such as *E. chinense*, permission from local and national governments must be acquired, via strict evaluation processes, prior to sample collection, which makes it difficult to conduct further research and collect samples. Moreover, direct collection of endangered species from foreign countries is largely prohibited by law, which explains the rareness, to date, of international-scale sample collections, and related research. Considering the difficulty in performing population genetic, phylogenetic, and demographical research on endangered species, the present study, which includes a well-organized research model, has great value for the global protection of *E. chinense*.

## Methods

### Sample collection and DNA extraction

In total, 103 individuals of *E. chinense* were collected from eight sampling sites across the western and southern coastal areas of South Korea; these covered the entire remaining habitats of *E. chinense* on the Korean Peninsula (Figs. 1B and S1; Table 1). Since this species is protected as an endangered species in South Korean government and the IUCN Red List, samples were collected after gaining permission from the Ministry of Environment, South Korea. The collected samples were immediately preserved in 95% ethanol immediately. Total genomic DNA was extracted from the foot tissue of *E. chinense* with the DNeasy Blood and Tissue Kit (Qiagen Co., USA) following the manufacturer’s protocol and then maintained at − 20 °C until further use. The efficiency of the genomic DNA extraction and the purity of the extracted DNA were examined with a spectrophotometer (Nanodrop Technologies, USA).

### PCR amplification and sequencing of COI

To amplify a partial fragment of mitochondrial *COI* gene of *E. chinense*, PCR was conducted using two universal primers: LCO1490 (5′-GGT CAA CAA ATC ATA AAG ATA TTG G-3′) and HCO2198 (5′-TAA ACT TCA GGG TGA CCA AAA AAT CA-3′). These primers, which were developed for invertebrates by Folmer et al.^35^, can be used for PCR amplification of a partial mitochondrial *COI* fragment, i.e. a DNA barcoding region, prior to sequencing. The PCR mixtures were prepared to a total volume of 50 μl containing 2 μl of 10 pM of each primer, 1 μl of 10 mM dNTP mix, 5 μl of 10× *Taq* DNA polymerase reaction buffer, 0.25 μl of 5 U/μl DiaSter *Taq* DNA polymerase, and 2 μl of total genomic DNA. Thermal cycling conditions were as follows: denaturation at 94 °C for 2 min, 35 cycles of 94 °C for 40 s, 52 °C for 1 min, and 72 °C for 1 min, and a final extension step of 72 °C for 7 min, which followed a cool down step at 4 °C. The PCR products were electrophoresed on 1% agarose gel including eco-dye and examined under UV light; they were purified using a QIAquick PCR Purification Kit (Qiagen Co., USA) and then sequenced using the primers applied in PCR and an ABI Prism 3730 DNA sequencer (PerkinElmer, USA) with a BigDye Termination Sequencing Kit (PerkinElmer, USA). The determined DNA sequences were manipulated in BioEdit v7.2.5^36^, and finally verified by visual inspection.

### PCR amplification and genotyping of microsatellite markers

A total of 30 microsatellite markers that have recently been developed for *E. chinense*^37^; these were tested using some representative *E. chinense* samples collected in the present study. The initial screenings discovered ten primer pairs showing clear and reproducible profiles; these were selected for subsequent genetic analyses of *E. chinense* populations (Table S1). A tailed PCR primer was produced by adding a M13 (− 21) universal sequence “tag” to the 5′ end of each forward primer as described by Schuelke^38^. The M13-forward oligonucleotide was labeled with the 6-FAM fluorescent dyes. PCRs were performed in a total volume of 20 μl consisting of 20 ng of template (gDNA), 10 mM dNTPs, 10 pmol of M13 modified forward primer, 10 pmol of each reverse primer and M13 primer fluorescently labeled with 6-FAM (Eurofins Genomics, France), with 10× incubation mix including MgCl_2_, 5 units HANLAB *Taq* DNA polymerase, and filled with distilled water. PCR cycling conditions comprised an initial denaturation phase at 94 °C for 5 min, followed by 34 cycles of 94 °C for 30 s, 54 °C for 30 s, and 72 °C for 30 s, and finally an extension step at 72 °C for 5 min. The tagged PCR product (0.2 μl) was added to 9.8 μl of Hi-Di formamide loading buffer, sized precisely using 0.2 μl of GeneScan 500 LIZ Size Standard (Applied Biosystems, USA) and subsequently run on an ABI 3730 × 1 automated sequencer (Applied Biosystems, USA). Allele size calling and genotypic analyses were performed using GeneMapper Software ver. 4.1 (Applied Biosystems, USA).

### Analyses of population genetic diversity and structure

To elucidate the level of genetic diversity and structure at the interpopulation and/or intrapopulation levels in *E. chinense*, the 140 obtained *COI* sequences were aligned with Clustal X^39^ (Data S1). The numbers of polymorphic sites and haplotypes, haplotype diversity, and nucleotide diversity were estimated for each population using the program DnaSP v.6.0^40^. AMOVA (analysis of molecular variance) was conducted in ARLEQUIN v.3.5^41^ to partition the genetic variance within and among populations. Pairwise *F*_ST_ values were calculated to assess the pattern of population differentiation. A neutrality test was performed by calculating Tajima's *D*^42^ and Fu's *Fs*^43^ via ARLEQUIN v.3.5^41^ to detect the historical existence of demographic expansion. To examine whether *E. chinense* has passed through recent expansion or frequent migration among neighbor demes, a mismatched distribution analysis was conducted based on the distribution of the observed pairwise nucleotide site differences and the expected values obtained assuming the populations of constant size or under the growth^15^. Based on the sequence alignment set of 58 *COI* haplotypes (Fig. S2; Data S2), unrooted and outgroup-rooted maximum likelihood (ML) trees were reconstructed using the IQ Tree web server (http://iqtree.cibiv.univie.ac.at) with the GTR + I + G model and 1000 bootstrapping replicates. Additionally, a phylogenetic network was generated via the neighbor-net algorithm^44^. Furthermore, a statistical parsimony haplotype network was constructed with a 95% connection limit using TCS v.1.2.1^45^; this was used to assess the genealogical relationship at the intraspecific level and to infer biogeography by constructing a *COI* haplotype network. To evaluate and visualize genetic differences among populations, principal coordinates analyses (PCoA) were conducted via DARwin v.6.0.9^46^, which ordinated genetic distance estimates calculated with the haplotype data used in this study.

Using the 10 microsatellite loci (Table S1) selected from the 30 microsatellite markers previously developed by Hyun et al.^37^, we reexamined the genetic diversity and structure of *E. chinense* populations in comparison to the results inferred from *COI* haplotype data. The PCR primers selected as microsatellite markers in the present study exhibited highly informative values of polymorphism information content (PIC > 0.5). Based on these microsatellite markers, genetic variability was estimated in terms of the number of alleles averaged over 10 loci (*N*_A_), observed heterozygosity (*H*_O_), and expected heterozygosity (*H*_E_) across populations and across loci using GenALEx v.6.5^47^. Furthermore, *F*_*IS*_ was calculated for each population and for each locus, based on Wright's F-statistics^48^. The genetic differentiation coefficient (*F*_*ST*_) was estimated using GenALEx v.6.5; additionally, tests for deviation from the Hardy–Weinberg Equilibrium between the microsatellite loci were performed using this software. To characterize the overall population genetic structure, we applied a Bayesian clustering approach implemented in STRUCTURE v.2.3.4^49^ which identifies the number of potential genetic clusters (*K*) in the dataset without a priori information on geographical or population identifiers. An admixture model was used with correlated allele frequencies applying burn-ins of 2 × 10^5^ and runs of 10^5^ repetitions for each value of *K* varying from 1 to 8. Three iterations were completed for each tested *K* value. The true number of *K* was identified based on the approach of Evanno et al.^19^ by applying Structure Harvester v.0.6.94^50^. In addition, we investigated isolation by distance (IBD) was examined by correlating the matrix of pairwise geographic distances between sampling locations with two correspondent matrices of genetic differentiation estimates: *F*_*ST*_ (estimated based on haplotype frequency) and *ф*_*ST*_ (considering the level of distance among haplotypes)^51^. Geographic distances were calculated as the shortest pathways along the coastal line.

### Covariance calculation and screening of possible G-quadruplex motifs in COI

From the multiple sequence alignment results of the *COI* haplotypes (Fig. S2), we searched for cases of concerted patterns of variation between different alignment positions. In performing this covariance calculation, we made pairwise gene sequence comparisons for every position. The base was checked for covariance with the frequency ratio in the multiple sequence alignment results, and this result was displayed on the network.

To screen for possible G-quadruplex-forming sequence motifs in *COI* (Fig. S1), we used the QGRS-Conserve program^52^, considering new types of G-quadruplex in addition to the canonical forms. The frequencies of nonoverlapping G-quadruplex-forming sequence motifs in the *COI* barcoding region were calculated using uninterrupted or interrupted by non-G-rich fragments not exceeding the maximum loop length (20 bp). This calculation process was repeated up to 10 times.

### Divergence time estimation based on COI

Divergence time estimation of the nodes on the phylogeny of *E. chinense* was conducted based on *COI* haplotype sequences via the BEAST 2.6.0^53^. The divergence time was estimated using the strict molecular clock algorithm under the calibrated-Yule tree prior. For the calibration point, the previously estimated age of the subfamily Carychiinae (from the family Ellobidae) was used as the basal node (60 Ma; normal distribution). The age of Carychiinae was given by Weigand et al.^54^, who among others reported that it originated no earlier than the beginning of the early Cenozoic (about 65 Ma)^14,54^. The best-fit nucleotide substitution model was selected by jModelTest^55^ as GTR + I + G. The posterior distributions of parameters were estimated using 1,000,000 MCMC generations with sampling every 1000 generations. Additionally, an effective population size value was determined in Tracer 1.7^56^. TreeAnnotator 2.6.0^57^ was used to produce a maximum clade credibility tree with a median height after removing the initial 25% of trees as burn-in. FigTree 1.4.2^58^ was used to visualize the topology of the resultant consensus tree.

## Supplementary Information

Supplementary Information 1.

Supplementary Information 2.

Supplementary Information 3.

Supplementary Information 4.

Supplementary Information 5.

## Data Availability

The sequence data obtained here are publicly available from NCBI GenBank under the accession numbers mentioned in the “Materials and methods”. The sequence alignment data sets of *COI* haplotypes, the result of covariate calculation, and the raw data of plausible G-quadruplex sequence motifs are available at Dryad Digital Repository, 10.5061/dryad.fbg79cnt4.
