# Supplementary material for: Characterization of metapopulation of Ellobium chinense through Pleistocene expansions and four covariate COI guanine-hotspots linked to G-quadruplex conformation
Source: Sci Rep. 2021 Jun 10;11:12239. doi: 10.1038/s41598-021-91675-5 (PMC8192772; doi:10.1038/s41598-021-91675-5)
Supplement: Supplementary file 1 — Supplementary Information 1. [file 41598_2021_91675_MOESM1_ESM.docx]

**Characterization of metapopulation of *Ellobium chinense* through Pleistocene expansions and four covariate *COI* guanine-hotspots linked to G-quadruplex conformation**

Cho Rong Shin, Eun Hwa Choi, Gyeongmin Kim, Su Youn Baek, Bia Park, Jihye Hwang, Jumin Jun, Hyun Jong Kil, Hyunkyung Oh, Kyungjin Lee, Sa Heung Kim, Jongrak Lee, Seung Jik Suh, Dong-min Park, Ho Young Suk, Yong Seok Lee, Young Sup Lee, and Ui Wook Hwang

*****To whom co-correspondence should be addressed Prof. Ui Wook Hwang (email: [*uwhwang1@gmail.com*](mailto:uwhwang1@gmail.com))

*Scientific Reports*

First manuscript submission: Jan. 22, 2021

First round revised version submission: May 03, 2021

**Table S1. Primer sequences, repeat motifs, and expected sizes for the 10 microsatellite loci selected for genotyping the endangered species *Ellobium chinense* inhabiting in South Korea and Japan.**

| **Loci** | **Primer sequences (5'-3')** | **Repeat motifs** | **Expected sizes (bp)** | **GenBank Acc. No.** | |
| --- | --- | --- | --- | --- | --- |
| ECHm13 | TTACTGGTGGTCTCCCCTGT | (GTT)_15_ | 372 – 474 | | KX881643 |
|  | ACGCTAACATGTGGTGCTCT |  |  |  |  |
| ECHm24 | GTGGATCCGTACCGTCCATC | (ATCC)_10_ | 212 – 276 | | KX881646 |
|  | AAAGGGGTAGTTGCCTTGGG |  |  |  |  |
| ECHm27 | GCCAGCTTTGTCTCTCCAGA | (GCTT)_11_ | 370 – 454 | | KX881647 |
|  | CAGTATAGACCAGCCCGCAG |  |  |  |  |
| ECHm28 | ACCCGCTCAAACTTTAGGCA | (ATCC)_12_ | 277 – 416 | | KX881648 |
|  | TGGTGTGCAGGTCTGTGAAA |  |  |  |  |
| ECHm31 | TCAACGGTGCTTTAGCCACT | (CTTT)_12_ | 418 – 502 | | KX881650 |
|  | CATCTGGTTTGGGGGTGTGA |  |  |  |  |
| ECHm35 | GTCACAGTGACCACTCCTGG | (GGAT)_15_ | 293 – 457 | | KX881653 |
|  | GCGTTTGACAAACAGCCAGT |  |  |  |  |
| ECHm36 | TTGGGCCAAGTCCAGTTGAA | (AAGG)_18_ | 287 – 506 | | KX881654 |
|  | TCCGATGCACCCACACATAA |  |  |  |  |
| ECHm40 | AGAAAGAACAGGCCCAGGTG | (ATGT)_20_ | 219 – 375 | | KX881656 |
|  | GATGGACAGGCAGGCAAGTA |  |  |  |  |
| ECHm41 | TTCCGTTACCATCGGGAAGG | (CATT)_21_ | 322 – 422 | | KX881657 |
|  | TTTCATCCCCCTGCCTTTGT |  |  |  |  |
| ECHm45 | CAAAGACCGTTTTCGTCGCA | (AAAC)_22_ | 234 – 338 | | KX881658 |
|  | TGTTGATCTCCACTGTGGCC |  |  |  |  |

**Table S2. The summary of 58 *COI* haplotypes observed from 140 *COI* sequences of *Ellobium chinense* along the nine populations in South Korea and Japan.**

| **Haplotypes** |  |  | **South Korea** | | | |  |  | **JK*** | **Total** | **GenBank Acc. No.** |
| --- | --- | --- | --- | --- | --- | --- | --- | --- | --- | --- | --- |
|  | **SC** | **BG** | **YG** | **HK** | **JB** | **BY** | **GY** | **HS** |  |  |  |
| ECH01 | 1 | 5 | 7 |  | 1 | 2 | 2 | 9 |  | 27 | MK696959.1, MK696968.1,  MK696967.1, MK696948.1 |
| ECH02 |  | 1 |  |  |  |  |  |  |  | 1 | MW265437 |
| ECH03 |  | 1 |  |  |  |  |  |  |  | 1 | MW265438 |
| ECH04 |  | 1 |  | 1 |  | 3 |  |  |  | 5 | MK696961.1 |
| ECH05 |  | 1 |  |  |  | 2 |  | 3 |  | 6 | MK696964.1 |
| ECH06 |  | 1 |  |  |  |  |  |  |  | 1 | MW265439 |
| ECH07 |  | 1 |  |  |  |  |  |  |  | 1 | MW265440 |
| ECH08 |  | 1 |  |  |  |  |  |  |  | 1 | MW265441 |
| ECH09 |  | 1 |  | 1 |  |  |  | 1 |  | 3 | MW265442 |
| ECH10 |  | 1 |  |  |  |  |  |  |  | 1 | MW265443 |
| ECH11 |  | 1 |  |  |  |  |  | 1 |  | 2 | MW265444 |
| ECH12 |  | 1 | 2 |  |  |  |  | 1 |  | 4 | MW265445 |
| ECH13 |  | 3 | 1 | 1 |  |  |  | 4 |  | 9 | MK696953.1, MK696952.1, MK696950.1 |
| ECH14 |  | 1 |  |  |  |  |  |  |  | 1 | MK696951.1 |
| ECH15 |  | 1 | 3 |  |  |  |  |  |  | 4 | MK696949.1 |
| ECH16 |  |  | 3 |  |  | 2 | 1 | 3 |  | 9 | MW265446 |
| ECH17 |  |  |  |  |  | 1 |  |  |  | 1 | MW265447 |
| ECH18 | 1 |  |  | 1 |  | 1 |  | 1 |  | 4 | MK696965.1, MK696946.1 |
| ECH19 |  |  |  |  |  | 1 |  |  |  | 1 | MW265448 |
| ECH20 |  |  | 3 | 3 | 1 | 1 | 2 | 6 |  | 16 | MK696957.1, MK696954.1, MK696963.1 |
| ECH21 |  |  |  |  |  | 2 |  |  |  | 2 | MK696962.1, MK696960.1 |
| ECH22 |  |  |  |  |  |  | 1 |  |  | 1 | MW265449 |
| ECH23 |  |  |  |  |  |  | 1 |  |  | 1 | MW265450 |
| ECH24 |  |  |  |  |  |  | 1 |  |  | 1 | MW265451 |
| ECH25 |  |  |  |  |  |  | 1 |  |  | 1 | MW265452 |
| ECH26 |  |  |  | 1 |  |  |  | 2 |  | 3 | MW265453 |
| ECH27 |  |  |  | 1 |  |  |  |  |  | 1 | MW265454 |
| ECH28 |  |  |  | 1 |  |  |  |  |  | 1 | MW265455 |
| ECH29 |  |  |  | 2 |  |  |  |  |  | 2 | MW265456 |
| ECH30 |  |  |  | 1 |  |  |  |  |  | 1 | MW265457 |
| ECH31 |  |  |  | 1 |  |  |  |  |  | 1 | MK696958.1 |
| ECH32 |  |  |  | 1 |  |  |  |  |  | 1 | MK696956.1 |
| ECH33 |  |  |  | 1 |  |  |  |  |  | 1 | MK696955.1 |
| ECH34 |  |  |  |  |  |  |  | 1 |  | 1 | MW265458 |
| ECH35 |  |  |  |  |  |  |  | 1 |  | 1 | MW265459 |
| ECH36 |  |  |  |  |  |  |  | 1 |  | 1 | MW265460 |
| ECH37 |  |  |  |  |  |  |  | 1 |  | 1 | MW265461 |
| ECH38 |  |  |  |  |  |  |  | 1 |  | 1 | MW265462 |
| ECH39 |  |  |  |  |  |  |  | 1 |  | 1 | MW265463 |
| ECH40 |  |  |  |  |  |  |  | 1 |  | 1 | MW265464 |
| ECH41 |  |  |  |  |  |  |  | 1 |  | 1 | MW265465 |
| ECH42 |  |  |  |  |  |  |  | 1 |  | 1 | MW265466 |
| ECH43 |  |  |  |  |  |  |  | 1 |  | 1 | MK696966.1 |
| ECH44 | 1 |  |  |  |  |  |  |  |  | 1 | MK696947.1 |
| ECH45 | 1 |  |  |  |  |  |  |  |  | 1 | MK696945.1 |
| ECH46 | 1 |  |  |  |  |  |  |  |  | 1 | MK696944.1 |
| ECH47 |  |  | 1 |  |  |  |  |  |  | 1 | MW265467 |
| ECH48 |  |  | 1 |  |  |  |  |  |  | 1 | MW265468 |
| ECH49 |  |  | 1 |  |  |  |  |  |  | 1 | MW265469 |
| ECH50 |  |  | 1 |  |  |  |  |  |  | 1 | MW265470 |
| ECH51 |  |  | 1 |  |  |  |  |  |  | 1 | MW265471 |
| ECH52 |  |  | 1 |  |  |  |  |  |  | 1 | MW265472 |
| ECH53 |  |  | 1 |  |  |  |  |  |  | 1 | MW265473 |
| ECH54 |  |  | 1 |  |  |  |  |  |  | 1 | MW265474 |
| ECH55 |  |  | 1 |  |  |  |  |  |  | 1 | MW265475 |
| ECH56 |  |  | 1 |  |  |  |  |  |  | 1 | MW265476 |
| ECH57 |  |  |  |  |  |  |  | 1 |  | 1  1 | MW265477 |
| ECH58 |  |  |  |  |  |  |  |  | 1 |  | KM281098.1 |
| **Total** | **5** | **21** | **29** | **16** | **2** | **15** | **9** | **42** | **1** | **140** |  |

*Japan. The localities of the populations refer to Table 1 and Figure 1.

**Table S3. Pairwise *F*_ST_ values estimated with 140 *COI* sequences from *Ellobium chinense* along the nine populations in South Korea and Japan.**

| **Populations** |  |  | **South Korea** | | | |  |  | **JK*** |
| --- | --- | --- | --- | --- | --- | --- | --- | --- | --- |
|  | **BG** | **BY** | **GY** | **HK** | **HS** | **JB** | **YG** | **SC** |  |
| BG | 0 |  |  |  |  |  |  |  |  |
| BY | -0.01429 | 0 |  |  |  |  |  |  |  |
| GY | -0.02505 | -0.01798 | 0 |  |  |  |  |  |  |
| HK | 0.00060 | -0.00744 | -0.03204 | 0 |  |  |  |  |  |
| HS | -0.01146 | -0.01389 | -0.02311 | -0.01664 | 0 |  |  |  |  |
| JB | -0.08155 | -0.00371 | **-0.21177** | -0.12690 | -0.12255 | 0 |  |  |  |
| YG | 0.00644 | 0.01001 | -0.03209 | 0.02426 | 0.00935 | -0.12712 | 0 |  |  |
| SC | -0.02042 | -0.04433 | -0.04716 | -0.01570 | -0.04828 | -0.13654 | -0.05606 | 0 |  |
| JK | 0.06400 | 0.01984 | -0.04082 | -0.03415 | 0.00697 | **0.09091** | 0.06293 | -0.06897 | 0 |

*Japan. The lowest and highest values are highlighted in bold. The localities of the populations refer to Table 1 and Figure 1.

**Table S4. Analysis of molecular variance (AMOVA) results performed with the *COI* sequences of 140 individuals from the nine populations of *Ellobium chinense*.**

| **Source of variation** | **Df**^1)^ | **Sum of squares** | **Variance components** | **Percentage of variation**^2)^ |
| --- | --- | --- | --- | --- |
| Among populations | 8 | 17.657 | -0.02692 | -1.05 |
| Within populations | 131 | 339.393 | 2.59079 | 101.05 |
| **Total** | **139** | **357.050** | **2.56387** | **100.00** |

1) Degree of freedom

2) The distribution of variation at a given level of hierarchy (among/within populations).

**Table S5. Genetic diversity estimated with the 10 microsatellite loci from 54 individuals representing the four populations of *Ellobium chinense* inhabiting South Korea and Japan.**

| **Populations** | ***N*** | ***N*_A_** | ***N*_E_** | ***H*_O_** | ***H*_E_** | ***F*_IS_** |
| --- | --- | --- | --- | --- | --- | --- |
| BG | 16 | 16.0 | 11.189 | 0.800 | 0.909 | 0.121 |
| YG | 10 | 11.4 | 8.537 | 0.810 | 0.875 | 0.078 |
| HK | 10 | 10.5 | 8.127 | 0.870 | 0.870 | 0.060 |
| HS | 18 | 15.7 | 10.126 | 0.881 | 0.881 | 0.103 |
| **Total/Mean** | **54** | **13.4** | **9.495** | **0.806** | **0.883** | **0.090** |

*N*: sample size (= the number of genotyped individuals), *N*_A_: the mean number of alleles, *N*_E_: the number of effective alleles, *H_O_*: observed heterozygosity, *H_E_*: expected heterozygosity, *F*_IS_: inbreeding coefficient (indicates populations with heterozygote deficit). The localities of the populations refer to Table 1 and Figure 1.

**Table S6. Pairwise *F*_ST_ and *R*_ST_ values obtained from the AMOVA test (analysis of molecular variance) using the 10 selected microsatellite loci, with 54 individuals representing the four populations of *Ellobium chinense* in South Korea and Japan.**

| **Populations** | **BG** | **YG** | **HK** | **HS** |
| --- | --- | --- | --- | --- |
| **BG** |  | 0.0018 | 0.0000 | 0.0000 |
| **YG** | 0.0054 |  | 0.0000 | 0.0141 |
| **HK** | 0.0089 | 0.0146 |  | 0.0000 |
| **HS** | **0.0120^*^** | 0.0077 | **0.0184^*^** |  |

Below diagonal: pairwise *F*st values between the populations, above diagonal: pairwise *R*st values between the populations. The localities of the populations refer to Table 1 and Figure 1. Statistically significant values are written in bold: *P < 0.05.

**Table S7. Analysis of molecular variance (AMOVA) results among the four populations of *Ellobium chinense* in South Korea and Japan, analyzed with the 10 selected microsatellite markers.**

| **Source of variation** | **Df**^1)^ | **Sum of Squares** | **Variance components** | **Percentage of variation**^2)^ |
| --- | --- | --- | --- | --- |
| Among populations | 3 | 17.894 | 0.02794 | 0.60 |
| Among individuals within populations | 50 | 261.384 | 0.60458 | 13.00 |
| Within populations | 54 | 217.000 | 4.01852 | 86.40 |
| **Total** | **107** | **496.278** | **4.65104** | **100.00** |

1) Degree of freedom

2) The distribution of variation at a given level of hierarchy (among populations/within populations).


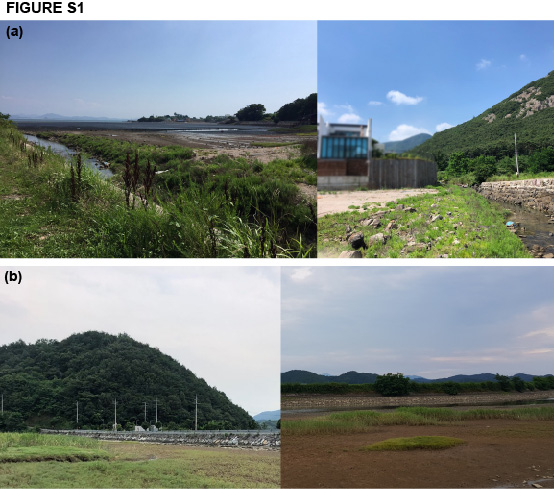


**Figure S1.** Photographs of the habitat landscapes of the land snail *Ellobium chinense* in South Korea. **(a)** The estuary of the Dongjin River, South Korea, where is close to salt marsh. *E. chinense* dwells between-stones and halophytes at this site. The photo was taken by CRS. in Byeonsan-myeon, Buan, Jeonbuk, South Korea. **(b)** The downstream region of the Seomjin River, South Korea, where is a little further from the shore. *E. chinense* mainly inhabits *Zoysia sinica* at this site. The photo was taken by CRS. in Jingyo-myeon, Hadong, Gyeongnam, South Korea. The photos were edited using Adobe Photoshop v.22.2 (<https://www.adobe.com>).


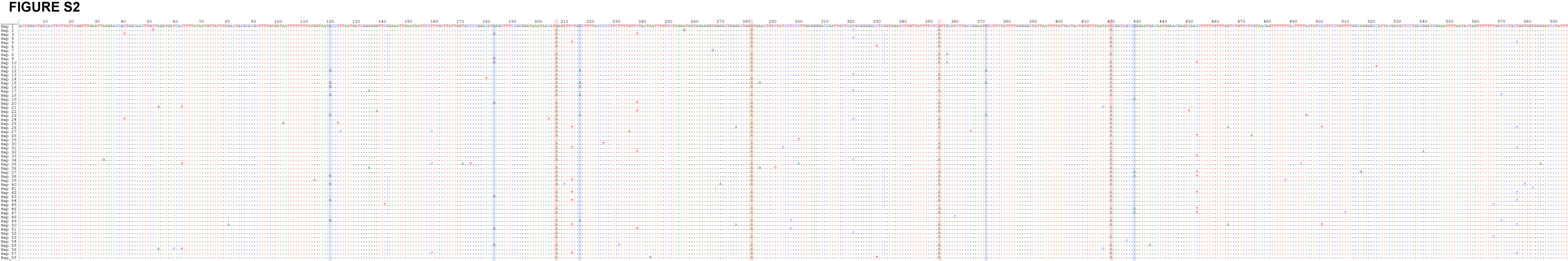


**Figure S2.** The sequence alignment of 58 *COI* haplotypes obtained from 140 *Ellobium chinense* individuals collected from South Korea and Japan. The dots mark sequences that are the same as those from the first line, ECH01. The four columns colored light pink indicate the four unidirectional stepwise A→G transition hotspots, which could be key sequences for classifying the haplotypes. The five sites partially colored with light aquamarine are additional A→G transitions, with a few exceptional G→A transitions. The sequence alignment set is displayed using BioEdit v7.2.5^3^ (https://bioedit.software.informer.com/7.2/).


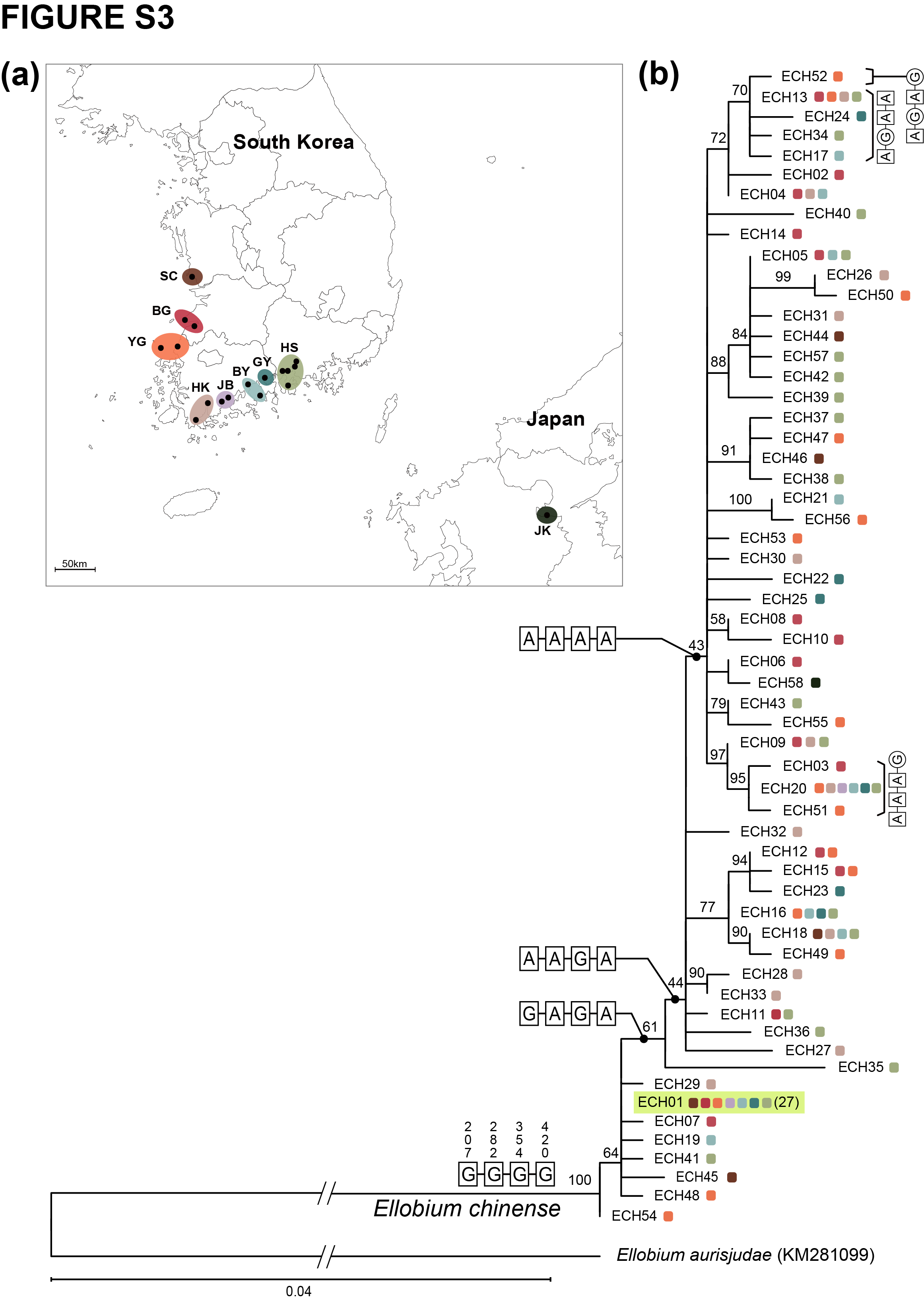


**Figure S3.** An outgroup-rooted maximum likelihood tree based on 58 *COI* haplotypes obtained from 140 *Ellobium chinense* individuals collected from South Korea and Japan. The numbers indicate bootstrap support values; when the value was <40, the branches collapsed. The color boxes on the map and the maximum likelihood tree indicate populations from sample collection sites. The dominant haplotype was ECH01, which was observed in 27 individuals of *E. chinense* from the seven collection sites at South Korea. The four genetic groups, namely A-A-A-A, A-A-G-A, G-A-G-A, and G-G-G-G, are depicted on the nodes of the maximum likelihood tree. *Ellobium aurisjudae* was used as an outgroup. The pictures were edited using Adobe Illustrator v.25.2 (<https://www.adobe.com>). The basic map is from a free map providing site (https://d-maps.com), which is modified with Adobe Illustrator v.25.2.


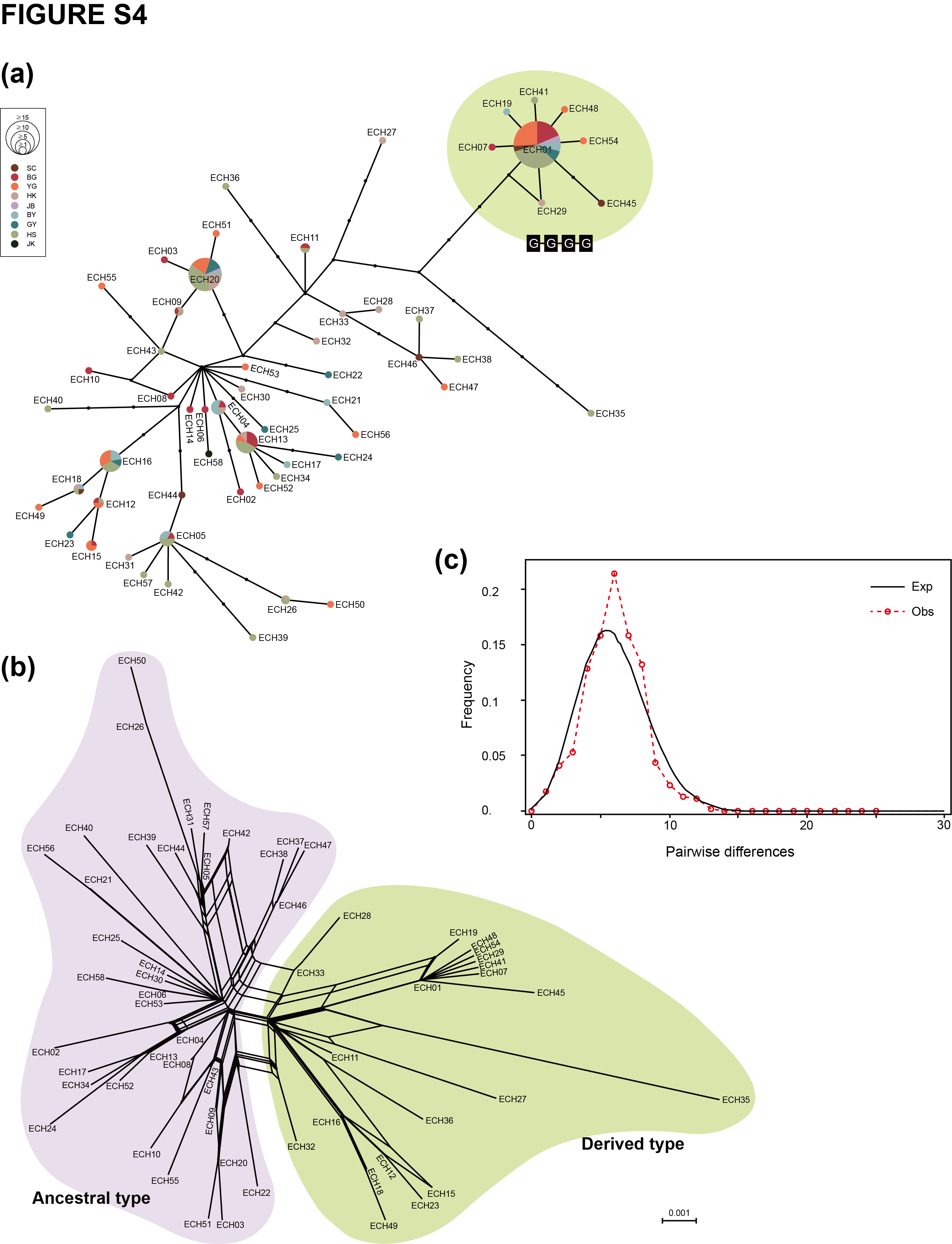


**Figure S4.** TCS network analysis, phylogenetic network analysis, and mismatch distribution analysis (MDA) based on 58 *COI* haplotypes obtained from 140 *Ellobium chinense* individuals collected from South Korea and Japan. **(a)** TCS network analysis results. Each of the crosshatched black dots represents one base pair difference between the haplotypes, and haplotype frequency is reflected by the size of the circle. The colors of the circles mark the collection sites at which the haplotypes were sampled. The *COI* haplotypes are listed with the GenBank accession numbers in Table S2. The most derived group of G-G-G-G is indicated by a light green-colored circle. **(b)** Phylogenetic network analysis results. The examined haplotypes were apparently closely related and exhibited metapopulation dynamics. The gray and light green areas indicate the ancestral genetic group and derived group, respectively. **(c)** MDA results. The unimodal graph implies that all the examined haplotypes form a single large metapopulation with frequent genetic flows. The pictures were edited using Adobe Illustrator v.25.2 (<https://www.adobe.com>).


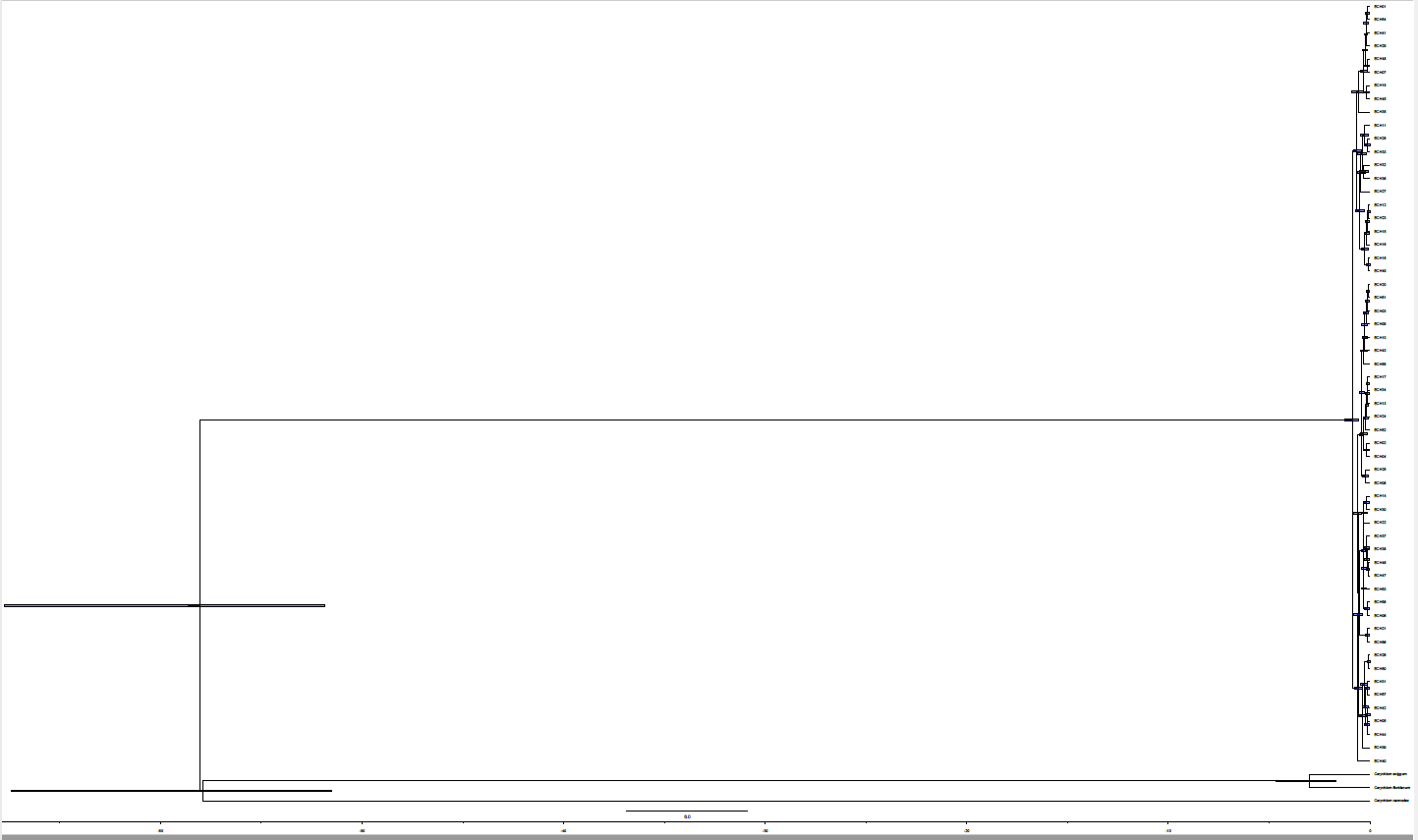


**Figure S5.** Molecular clock analysis based on 58 *COI* haplotypes obtained from 140 *Ellobium chinense* individuals collected from South Korea and Japan. Analysis was conducted in BEAST 2.6.0. Refer to Fig. 4b for the detailed divergence times of the *COI* haplotypes of *E. chinense*. Outgroups and calibration points are described in the Materials and Methods.

**Data S1.** The alignment set of the 140 *COI* sequences of *Ellobium chinense* inhabiting South Korea and Japan, among which 113 *COI* sequences were obtained from the present study and 27 *COI* sequences were retrieved from the GenBank database.

**Data S2.** The sequence alignment set of the 58 *COI* haplotypes observed in 140 individuals of *Ellobium chinense* inhabiting South Korea and Japan, which are from the results of the present study and retrieved ones from the GenBank database.

**Data S3.** Raw data obtained from the covariance tests performed for constructing a covariance network performed in this study. The four *COI* hotspots reveal high covariance values in common. The results are summarized and depicted as a network in Figure 2b.

**Data S4.** Putative GQRS-Conserve motifs capable of G-quadruplex structures on CDS of the *COI* barcoding region of *Ellobium chinense* inhabiting South Korea and Japan. The green boxes mean the selected plausible G-quadruplex motifs shown in Figure 2c.
